# Fabrication, Simulation, and Mechanical Characterization of Curcumin-Loaded PVA/PVP Microneedle Arrays Using Custom 3D-Printed Molds

**DOI:** 10.3390/polym18151912

**Published:** 2026-08-04

**Authors:** Bryan Angelo S. J. Basa, Charlize Dawn Z. Batin, Izabelle Nisha Maxine D. Chan, Adrian Ray B. Gabay, John Ray C. Estrellado, Ron Gilbert R. Rallos, Mary Stephanie S. Carranza, Mark Jefferson U. Lim, Jubert C. Marquez, Joseph Rey H. Sta Agueda

**Affiliations:** 1 Department of Manufacturing Engineering and Management, De La Salle University, Manila 1004, Philippines; bryan_basa@dlsu.edu.ph (B.A.S.J.B.); charlize_batin@dlsu.edu.ph (C.D.Z.B.); izabelle_chan@dlsu.edu.ph (I.N.M.D.C.); adrian_gabay@dlsu.edu.ph (A.R.B.G.); john.ray.estrellado@dlsu.edu.ph (J.R.C.E.); 2 Center of Engineering and Sustainable Development Research, De La Salle University, Manila 1004, Philippines; ron.rallos@dlsu.edu.ph (R.G.R.R.); mary.stephanie.carranza@dlsu.edu.ph (M.S.S.C.); mark.jefferson.lim@dlsu.edu.ph (M.J.U.L.); 3 Department of Chemical Engineering, De La Salle University, Manila 1004, Philippines; 4 Cardiovascular and Metabolic Disease Center, Paik Hospital, Inje University, Busan 47392, Republic of Korea; jubert.marquez@dlsu.edu.ph

**Keywords:** Microneedle arrays, curcumin, 3D printing, finite element analysis

## Abstract

Microneedle (MN) arrays offer a novel and minimally invasive platform for transdermal drug delivery. This study presents an approach for the design and fabrication of MN array models for biomedical applications using custom 3D-printed micro-molds. Material analysis of the polyvinyl alcohol (PVA) and polyvinylpyrrolidone (PVP) matrix in a 3:1 weight ratio was conducted under varying geometric configurations, curcumin (CUR) dosages, and target penetration depths of 300 to 500 μm. Computational simulation using computer-aided design (CAD) and finite element analysis (FEA) on ANSYS (Canonsburg, PN, USA) measured for total deformation, stress distribution, insertion pressure, and factor of safety. Elimination criteria were applied, narrowing down to specific models that were experimentally validated through material formulation, micro-molding, and material characterization. The selected MN models were analyzed by insertion and penetration efficiency testing on porcine skin. The results showed that higher CUR concentrations reduced mechanical strength and Young’s modulus, while mid-range dosages (2–6 mg) combined with optimized geometric spacing produced MNs with maintained structural integrity and effective performance. Conical microneedles demonstrated the most favorable balance of mechanical stability, controlled swelling behavior, and high insertion efficiency. The study recommends this approach as a feasible and reproducible method for localized wound-healing applications.

## 1. Introduction

Microneedle (MN) arrays have emerged as a minimally invasive and patient-friendly platform for transdermal drug delivery, offering a practical alternative to conventional methods such as oral and intravenous administration, which are often limited by reduced bioavailability, invasiveness, and systemic side effects [1,2]. By penetrating the stratum corneum, MNs create microchannels that bypass the skin’s natural barriers [3], enabling localized and controlled delivery of therapeutic agents. This targeted administration enhances drug bioavailability, reduces systemic exposure, and improves patient adherence—an important consideration for treatments requiring repeated dosing or long-term management. In wound-healing applications, MNs offer additional advantages by stimulating the skin and enhancing micro-injury-induced cellular responses, promoting tissue proliferation and supporting wound debridement and healing [4]. Compared to conventional wound-care strategies, MNs can provide sustained release of therapeutics directly at the wound site, aiding extracellular matrix remodeling and accelerating overall healing [5].

Beyond wound care, MNs have been extensively explored in other medical and diagnostic fields, including vaccine delivery [6,7], diabetes management [8,9], and cosmetic dermatology. Kim et al. highlighted the use of hollow MNs for vaccination and solid MNs for cosmetic therapies [10], while Lee et al. demonstrated the efficacy of polymer-based MNs for transdermal delivery of hispidin in skin rejuvenation [11]. Dissolvable MNs, in particular, present unique benefits because they degrade safely in the skin, eliminating sharp waste and reducing infection risk. However, their mechanical strength, insertion efficiency, and drug-release behavior are highly dependent on needle geometry, length, and material properties [12,13,14]. Despite increasing interest, reproducible design parameters—especially needle shape and optimal insertion depth of 300–500 μm—remain insufficiently established for dissolvable MNs intended for wound-healing applications.

Curcumin (CUR) is a promising candidate for MN-mediated wound treatment due to its anti-inflammatory, antioxidant, antimicrobial, and pro-regenerative properties. It promotes fibroblast proliferation, collagen deposition, and modulation of the wound microenvironment, making it suitable for both acute and chronic wounds [15,16,17]. However, CUR’s hydrophobicity and thermal sensitivity limit its compatibility with hydrophilic polymers such as polyvinyl alcohol (PVA) and polyvinylpyrrolidone (PVP). Encapsulation via hydrophilic carriers may improve its solubility and stability within these matrices [18,19]. Despite its therapeutic potential, the translation of CUR-loaded MNs to commercial products remains limited due to challenges in achieving reproducible large-scale manufacturing. Variability in polymer formulation, curing and drying conditions, and microfabrication precision (e.g., printing or casting) continues to hinder uniform MN production. CUR has been widely studied in MN-based wound-healing systems due to its broad therapeutic window (0.092–41.6 µg/mL) and compatibility with variable MN depths (8–500 µm) [20,21,22,23]. Its integration into hydrogel or bilayer MN formulations supports sustained release and improved structural stability, distinguishing it from other dissolving MN systems such as those incorporating collagen [24,25] or ibuprofen [26,27]. Moreover, CUR’s low cytotoxicity at therapeutic doses positions it as a safer alternative to high-concentration steroids or antibiotics commonly used in wound care [28].

Given the current gaps in MN design standardization, there is a strong need to evaluate the mechanical performance, structural fidelity, and fabrication reproducibility of CUR-loaded dissolvable MNs. This study aims to develop dissolvable PVA/PVP MN arrays containing curcumin for localized wound-healing applications. Specifically, the work evaluates the mechanical properties of CUR-loaded MNs, compares needle geometries (bullet, pyramidal, and conical), identifies optimal dimensions and spacing capable of achieving insertion depths of 300–500 μm, and fabricates MN arrays with varying curcumin dosages. The scope is confined to materials engineering, device fabrication, and structural performance, excluding biological efficacy or degradation studies. The goal is to establish reproducible MN design considerations essential for advancing dissolvable MN technologies in wound management.

## 2. Materials and Methods

### 2.1. Materials

Polyvinyl Alcohol (AR-grade, Dalkem, Quezon City (QC), Philippines), polyvinylpyrrolidone (AR-grade, Dalkem, QC, Philippines), and phosphate-buffered saline (AR-grade, Sigma Aldrich P4417) were obtained from commercial sources. Sorbitol (AR-grade, CAS No. 50-70-4) and curcumin (CUR) (AR-grade) were sourced from the Biomaterials and Tissue Engineering Laboratory (BIMATEL).

### 2.2. Formulation of PVA-PVP-CUR Polymer Matrix

The PVA-PVP solution was done by preparing a 7% (*w*/*v*) PVA base solution by gradually dissolving PVA powder in hot distilled water under continuous stirring at 1000–1500 rpm and at 80 °C for 1 h rpm [20,29,30]. This was followed by the preparation of a 20% (*w*/*v*) PVP solution at room temperature for 30 min. The solutions were mixed at a 3:1 (*v*/*v*) PVA:PVP ratio and stirred with a glass stirring rod at room temperature (25 °C) until homogeneous, a ratio chosen for its enhanced mechanical strength and swelling properties [31,32]. Sorbitol was added at 5% *w*/*w* relative to the total dry weight of the polymers [33]. The PVA-PVP-CUR polymer matrix was synthesized by preparing the PVA-PVP solution followed by the addition of the CUR solution. Once both solutions were ready, they were combined, and the mixture was processed to fabricate the polymer films. All samples were prepared according to the formulations summarized in Table 1 where each formulation was delegated a corresponding CUR dosage (µg/mL) (Equation (1)) and CUR patch dosage (µg) (Equation (2)) [20,22,34].

(1) $$C_{D}(\mu g/mL)=\frac{C_{T}(mg)\;\cdot \;1000}{\;V_{T}\;(mL)}$$

(2) $$C_{D_{patch}}\;(\mu g)=C_{D}\;(\mu g/mL)\;\cdot \;V_{P}\;(mL)\;$$

### 2.3. Characterization of Physiochemical Properties

Swelling behavior was examined according to ASTM F2214-23 [35] by immersing polymer films (F1–F6) in 1% PBS and recording mass changes over 60 min. The swell ratio (SR) was calculated using the initial dry mass (M_0_) and swollen mass (Mₜ) (Equation (3)). This analysis provided insight into fluid uptake and potential dissolution behavior of the MNs, where higher SR values indicated greater water absorption but also possible reductions in structural stability. The swelling behavior of MN formulations F1–F6 was assessed over 60 min to evaluate the impact of curcumin (CUR) concentration on water uptake and matrix stability. All formulations absorbed water, with swelling peaking at 15 min.

Compositional characterization was performed using Fourier-transform infrared (PerkinElmer, Spectrum^TM^ 3) (Waltham, MA, USA) spectroscopy in accordance with ASTM E1252 [36] to confirm the presence of PVA, PVP, SOR, and CUR and to verify the removal of residual solvents. Spectra were collected from 500 to 4000 cm^−1^ at a resolution of 4 cm^−1^ and processed in OriginPro (v10.1, 2024) (Northampton, MA, USA).

(3) $$Swell\;Ratio\;(SR)=\frac{M_{t}-M_{0}}{M_{0}}$$

### 2.4. Simulation and Synthesis of Reverse Silicone MN Mold

**Figure 1:**
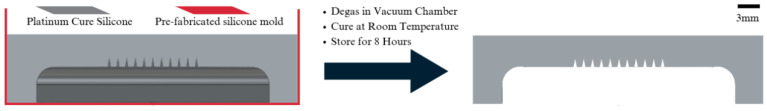
Preparation of 3D-printed male mold.

MN geometries were modeled in Autodesk Fusion 360 to compare mechanical behavior and insertion performance across three designs: conical, pyramidal, and bulleted (Figure S1), all of which were selected for their structural and functional considerations. All geometries followed a 1:3 height-to-base aspect ratio to ensure consistent penetration mechanics, with a 4:3 height-to-spacing ratio to maintain inter-needle support (Table S2) [37]. Customized male molds (Figure 1) were printed with an Elegoo Saturn 4 printer (Shenzhen, China) with a resolution of 19 × 24 µm using eSun eResin-PLA Pro. The printing process was conducted with a layer height of 25 µm and a printing angle of 60° to achieve high precision and structural integrity. Arrays of 11 × 11, 13 × 13, and 15 × 15 MNs were created to undergo finite element analysis (FEA) to evaluate the effects of needle quantity and spacing on stress distribution, structural integrity, and delivery potential (Table S4) [38,39]. The selection, based on total deformation, equivalent stress, and factor of safety (FOS) thresholds would be consolidated through structural simulation software, ANSYS (ANSYS r2, 2024). Factor of safety (FOS) was calculated by determining yield strength of the materials stress limit before plastic deformation (MPa) divided by the maximum equivalent stress (σ) (von Mises) obtained from the simulation (MPa) (Equation (4)). Only the MNs that satisfied all mechanical requirements were considered for fabrication and experimental validation.

(4) $$Factor\;of\;Safety\;(FOS)=\frac{Yield\;Strength}{\sigma_{max}}$$

### 2.5. Fabrication of CUR-Loaded PVA-PVP MNs

Formulated MN solutions were then cast into the female molds, with 2 mL of formulation added to each mold to fully fill the cavities. The filled molds were degassed under −0.8 BAR pressure for 4 min to eliminate trapped air and dried at 30 °C for 4 h to solidify the MNs (Figure 1). No anti-adhesive agents were used. After drying, the MNs were demolded, trimmed to a final array size of 20 × 20 mm, and stored at room temperature for subsequent prototype testing.

### 2.6. Morphological and Mechanical Structure Analysis of MNs

Scanning electron microscopy (JEOL JSM IT500HR) (Tokyo, Japan) was performed on gold-sputtered samples (JEC-3000 FC) (Tokyo, Japan) to examine microneedle morphology and surface integrity. Both blank and curcumin-loaded microneedles were imaged at 500× magnification following ASTM E2809-22 [40]. Tip geometry, surface smoothness, and overall structural uniformity were assessed, with some samples imaged at a tilted angle to better visualize tip structure. To assess their mechanical strength, MN patches of different compositions were compression-tested using the UniVert, CellScale Biomaterials Testing (Waterloo, ON, Canada). MNs were placed tip-up on a rigid platform, and a sensor descended at 0.08 mm/s until contact. Force-displacement curves were recorded, and the compressive force at 0.8 mm displacement was compared among samples.

### 2.7. Evaluation of MN Performance

The MN insertion depth was assessed using a compound microscope with a set magnification of 10×. The insertion depth of each MN model was measured before and after penetration using a graticule slide, which is marked with grid lines and provided the models with a measurement reference. Each fabricated MN was measured and crosschecked with their supposed initial height of 1500 µm. A 500 µm reference was set per four boxes in the graticule slide grid, making each individual grid line equivalent to 100 µm. Measurements were done in triplicate where L_i_ represents the initial length of the microneedle before insertion (μm), and L_f_ represents the final length of the microneedle remaining after removal from the skin (μm) (Equation (5)). Insertion efficiency was measured using (Equation (7)).

Penetration efficiency (%) of MN arrays was tested on PBS-soaked pork skin prepared by removing hair and subcutaneous layers. After insertion and a 15 min dissolution period, the skin was stained with methylene blue then washed with isopropyl to remove excess stains to highlight puncture sites. The number of visible holes (*N_p_*) was compared to the total number of MN within the array (*N*_t_) [39] (Equation (6)).

(5) $$Insertion\;depth={\;L}_{i}-L_{f}$$

(6) $$Penetration\;Efficiency\;(PE)\;(\%)=(\frac{N_{p}}{N_{t}})\times 100\;$$

(7) $$Insertion\;\;Efficiency\;(EE)\;(\%)=(\frac{L_{p}}{L_{t}})\times 100$$

## 3. Results and Discussion

### 3.1. Chemical Structure Analysis of Polymer-Drug Formulation

**Figure 2:**
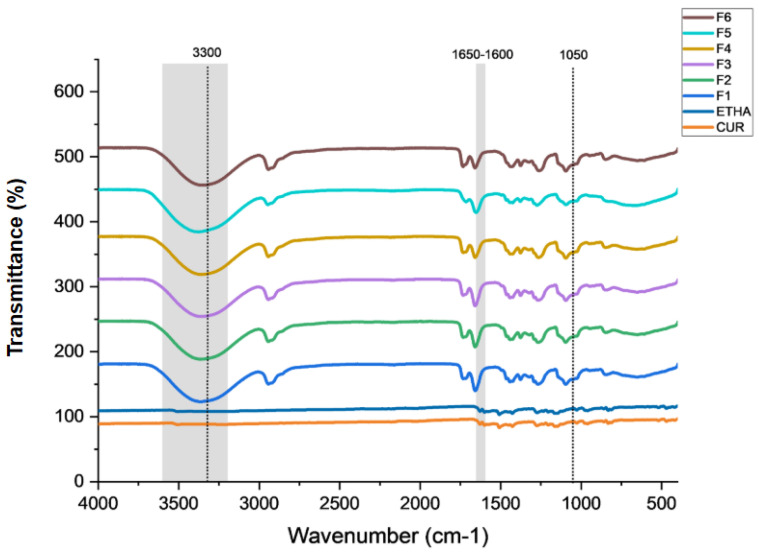
FT-IR spectra overlay of samples F1–F6 vs. CUR.

The formulation spectra are dominated by the functional groups present in PVA, PVP, and sorbitol (Figure 2). Although F1 contains no curcumin (negative control), its spectrum closely resembles F2–F6 due to strong signals contributed by the bulk polymer present (Table S1). In F2–F6, a minor increase in intensity in the ~1600 cm^−1^ region and a broader O–H region around ~3300 cm^−1^ (shadow regions) indicate curcumin incorporation. The absence of ethanol peaks (dashed lines ~3300 and ~1050 cm^−1^) confirms complete solvent removal. Overall, the spectra show consistent polymer structure and clear compositional differences in curcumin-loaded formulations.

### 3.2. Mechanical Tests

Formulation F1, with no curcumin (0 mg), recorded the highest ultimate tensile strength (UTS) (4.8133 MPa), Young’s modulus (2.8909 MPa), and yield strength (2.3526 MPa), but the lowest strain at maximum force (166.5%), indicating a more rigid and mechanically strong structure. In contrast, formulation F6 (10 mg CUR) exhibited the lowest UTS (2.4833 MPa), Young’s modulus (0.9931 MPa), and yield strength (1.5116 MPa) but the highest strain (250.07%), suggesting greater flexibility and elongation before failure. The tensile mechanical properties demonstrate a decrease in ultimate tensile strength (UTS), Young’s modulus, and yield strength as the curcumin content increases (Table 2). This trend suggests that higher drug loading reduces the mechanical integrity of the MN material. Additionally, with higher curcumin dosage, strength and stiffness is reduced but flexibility increases. Increase in ductility (increase in strain at F_max_) suggests a softer and more deformable MN at higher CUR concentrations. The negative correlation between curcumin dosage and MN yield strength can be possibly attributed to CUR acting as a plasticizing agent. This is consistent with the previous literature on polymer-based microneedles citing a reduction in tensile strength and stiffness as the curcumin content increases. This also reinforces the importance of considering the impact of drug concentration on the structural integrity and therefore, efficiency of the developed MN [20,39,41,42].

### 3.3. Swelling Test

**Figure 3:**
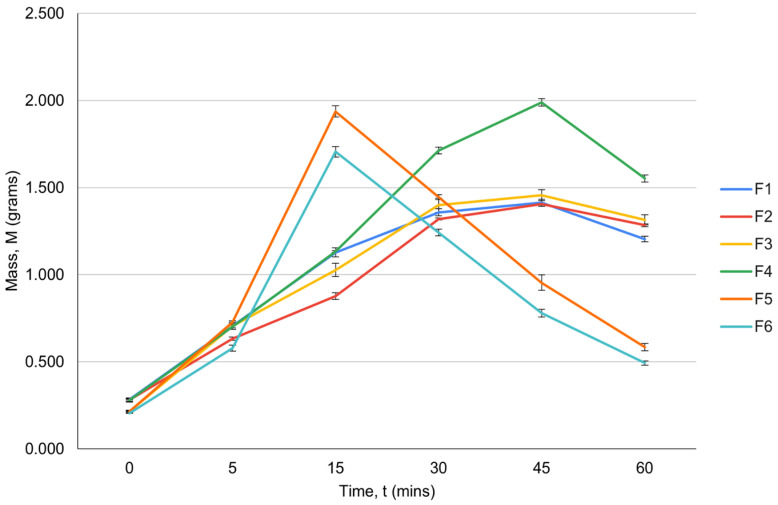
Graphical representation of F1 to F6 swell behavior. One-way ANOVA confirmed a significant effect of CUR content on swelling (F = 57.88–1549.62, *p* < 0.05).

Higher CUR concentrations (F5–F6) showed the highest initial swelling ratios, with values of 7.33 and 8.01 for both formulations, respectively, followed by sharp declines, indicating structural collapse due to excessive water uptake. The sudden uptake and release of water may be attributed to CUR reducing intermolecular polymer interactions, causing fast swelling. Once the MN with higher CUR dosages had over-swollen, it mechanically collapsed, leading to bulk material release [20]. In contrast, lower CUR formulations (F1–F3) demonstrated stable, sustained swelling, with F3 (4 mg CUR) reaching SR_45_ = 5.77. Moderate CUR content (F4: 6 mg) achieved the highest controlled swell (SR_45_ = 6.13) without degradation (Figure 3). Visual analysis of PBS coloration supported these findings, with higher CUR doses showing burst release, while lower doses favored gradual diffusion, suitable for sustained drug delivery.

### 3.4. Determination of Optimal MN Dimensions

Among all geometries and curcumin dosages, the conical MN design demonstrated the best mechanical performance and structural integrity (Figure S2, Table S2). More specifically, the conical models with an 11 × 11 array at 0–6 mg CUR and a 13 × 13 array at 0 mg and 2 mg CUR met all established elimination criteria (Table S3). The FEA simulations of conical MN arrays demonstrated trends that were consistent with the tensile behavior of the polymer formulations (Figure S3–S5, Table S4–S6). As CUR loading increased, total deformation rose (0.29 to 0.49 mm), equivalent stress intensified (0.62 to 3.56 MPa depending on array size), and the factor of safety decreased (Table 3, Figure S6). These changes indicate a heightened susceptibility to bending or mechanical failure during insertion, which reflects the experimentally observed decline in stiffness and strength. The strong alignment between tensile and simulation data confirms that the mechanical weakening caused by higher CUR content directly influences MN performance (Figures S7–S12, Tables S7–S9). Formulations with higher stiffness (e.g., F1–F2) generated lower deformation and higher safety margins in FEA, suggesting they are better suited for reliable penetration. In contrast, more ductile formulations (F5–F6), although flexible, exhibited excessive deformation that may compromise insertion depth and drug delivery efficiency.

### 3.5. Penetration Efficiency and Compression Property

**Figure 4:**
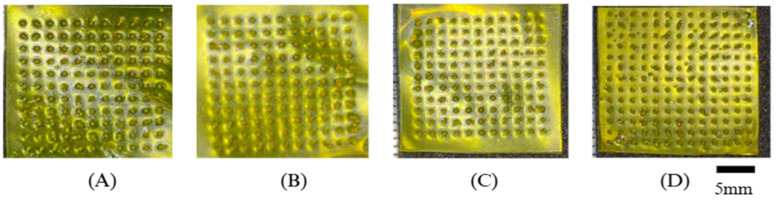
Fabricated dissolvable PVA-PVP microneedle prototypes (models (**A**–**D**) corresponding to CUR loaded (mg) and resulting FEA data in Table 3).

**Figure 5:**
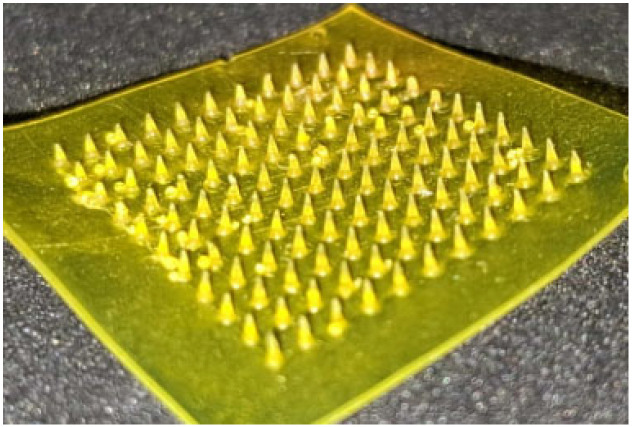
Isometric view of fabricated microneedle prototype model A.

**Figure 6:**
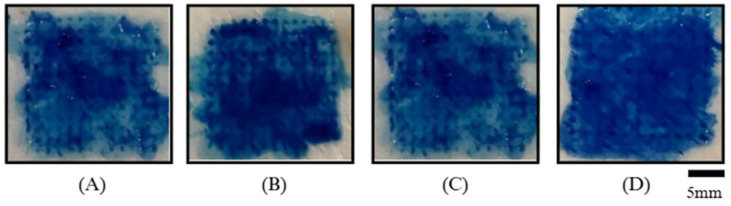
Pork skin after methylene blue staining showing microneedle penetration sites for arrays (**A**–**D**).

None of the tested MN models exhibited the “bed of nails” effect, a phenomenon where MNs fail to puncture the skin effectively due to a lack of spacing (Figure 4). The absence of this issue across all models suggests that the MNs were able to generate sufficient spacing and localized pressure to penetrate the skin, further supporting their effectiveness (Figure 5). The analysis showed that the number of successful insertions varied significantly across models A through D, indicating that MN design strongly influences skin penetration efficiency (Figure 6,  Figures S13 and S14). A one-way ANOVA revealed statistically significant differences in insertion efficiency among the four MN models (F = 110.34, *p* < 0.001). *p*-values less than 0.05 were considered significant.

**Figure 7:**
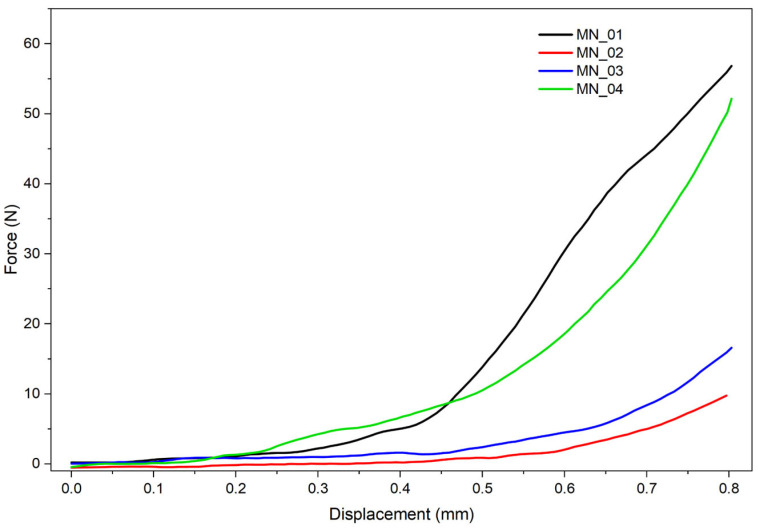
Representative compression force-displacement curves of MN patches.

MN patches must withstand compressive forces during skin penetration. Since MNs are designed to penetrate the skin without mechanical failure, resistance to compression at a defined displacement is a relevant indicator of their ability to maintain structural integrity during insertion. As shown in Figure 7, MN_01 (MN without SOR) exhibited the highest compressive force with 56.38 N, followed by MN_04 (high-dose CUR-loaded MN) with 51.66 N, MN_03 (low-dose CUR-loaded MN) with 16.29 N, and MN_02 (MN with SOR) with 9.56 N. The compression testing results are consistent with the trends observed in the tensile mechanical properties. Formulations with higher tensile strength, Young’s modulus, and yield strength demonstrated greater resistance to compressive forces, as seen in MN_01, which exhibited the highest compressive force. All MN patches exceeded the minimum force requirement of 21 mN per needle for skin penetration (Table 4) [43].

### 3.6. Insertion Analysis

The measured values were recorded and are summarized in Table 5. A one-way ANOVA revealed statistically significant differences in insertion depths among the four tested MN models (F = 88.81, *p* < 0.001). *p*-values less than 0.05 were considered statistically significant. With height held constant within grouped designs, the analysis demonstrated that penetration depth varied significantly across models A to D (one-way ANOVA, *p* < 0.001).

### 3.7. Scanning Electron Microscopy (SEM) Analysis

**Figure 8:**
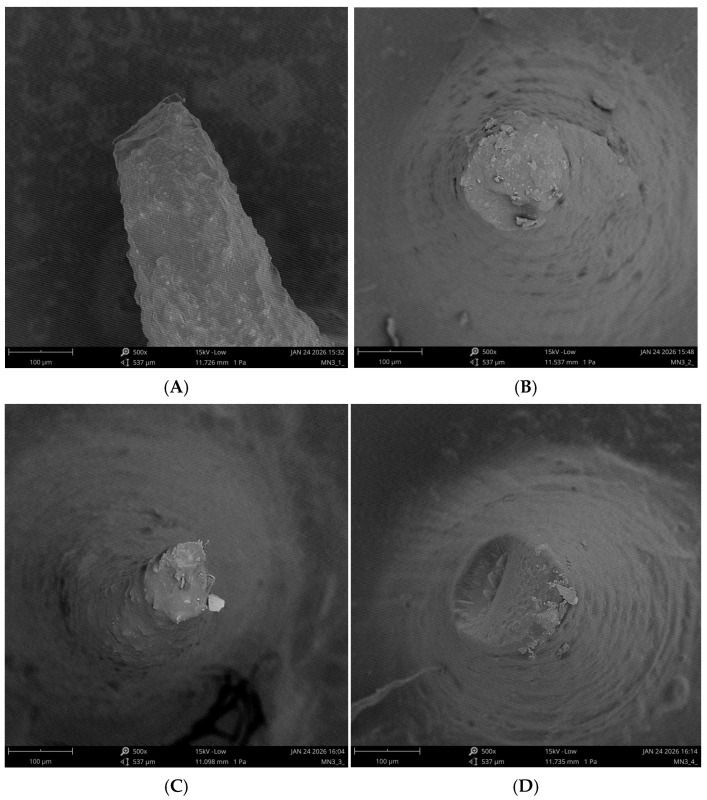
SEM images of MNs at 500× magnification under different formulations: (**A**) MN without SOR; (**B**) MN with SOR; (**C**) low-dose CUR-loaded MN; and (**D**) high-dose CUR-loaded MN.

The drug-free MN surface exhibited a uniformly smooth appearance without noticeable cracks or deformities, which is consistent with prior findings where optimized fabrication parameters led to homogeneous polymer surfaces [30]. In contrast, the surface of the drug-filled MNs displayed distinct irregularities, including uneven regions and minor surface roughness, potentially due to the distribution of the drug within the polymer matrix (Figure S15). SEM view of a slightly tilted singular MN needle of the drug-filled MN array was captured, highlighting the MN geometry and tip sharpness (Figure 8). Similar geometry was reported by Krieger et al., where high-fidelity mold designs led to sharp microneedle tips and consistent dimensions [38]. The MN appeared well-formed, demonstrating a conical shape, confirming successful mold replication. However, small air bubbles were also observed within the structures, likely resulting from rapid drying oven conditions during fabrication. These internal irregularities could potentially impact mechanical strength. Small surface bumps were also detected, likely resulting from air bubbles trapped within the female silicone molds.

### 3.8. CUR Release Profile

**Figure 9:**
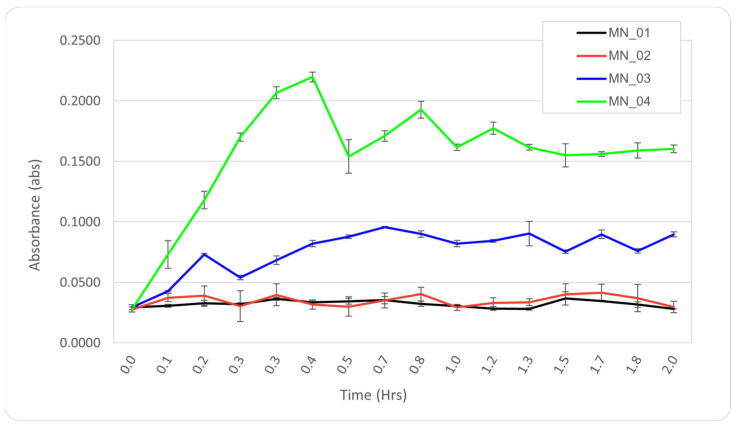
Curcumin (CUR) release profile of MN patches with sorbitol (5% *w*/*v*).

The release profile of CUR (Figure 9) exhibited a sustained and concentration-dependent release profile, with distinguishably higher cumulative release from patches loaded with 10 mg CUR (MN_04) versus 2 mg CUR (MN_03). Additionally, across all time points, the higher CUR-loaded MN demonstrated a consistent elevated release, with a pronounced increase toward the later stages of the assay, suggesting a bulk release in CUR and its passive interaction with the patch matrix in contrast to the lower CUR-loaded MN, where a more moderate and relatively stable release profile is observed. This was indicative of a better retention within the patch matrix. The presence of sorbitol was included in the analysis to determine its effect where it can be observed that little to no difference was detected (Figure S16). Taken together with independent data showing reduced tensile strength and stability in sorbitol-rich patches, these results suggest that higher sorbitol content may promote faster curcumin release by weakening matrix integrity, potentially at the expense of mechanical robustness and controlled delivery performance.

## 4. Conclusions

This study successfully developed stereolithography-assisted micro-molded microneedle (MN) arrays for curcumin loading by integrating formulation optimization, mechanical testing, swelling analysis, finite element modeling, and insertion performance. Mechanical characterization showed that increasing CUR concentration substantially reduced polymer strength: ultimate tensile strength decreased from 4.81 MPa (F1) to 2.48 MPa (F6), accompanied by decreases in Young’s modulus and yield strength, while strain at failure increased from 166% to 250%. These results indicate that higher CUR loading softens the matrix and weakens structural integrity. Swelling behavior supported this trend, with F5 and F6 displaying rapid mass gain followed by sudden mass loss, suggesting CUR-induced osmotic imbalance and compromised crosslinking. In contrast, F1–F4 exhibited stable swelling profiles, confirming that excessive CUR disrupts polymer stability needed for reliable MN performance. Finite element analysis further demonstrated that increasing CUR increased deformation and reduced safety factors (e.g., 0.294 mm to 0.488 mm deformation in the 11 × 11 geometry), narrowing viable designs. Only four MN models met mechanical and safety criteria: three 11 × 11 arrays (A–C) and one 13 × 13 array (D), all using the mechanically stable F2 formulation. Insertion and penetration tests validated these selections. Models A and C achieved 96.69% puncture efficiency with insertion depths of 1220 µm and 1201 µm, exceeding the clinically relevant 300–500 µm range. Furthermore, the compression test results confirm that the MN patches possess adequate mechanical robustness for skin insertion. Although the 13 × 13 design (Model D) reached 94.67% puncture efficiency, its reduced depth (960 µm) reflected higher stress concentrations predicted in simulations. Optimal performance corresponded to an interspacing of 1125 µm and intraspacing of 1625 µm, which balanced mechanical strength and penetration capability. SEM analysis revealed smooth, homogeneous surfaces in drug-free MNs, whereas drug-loaded MNs showed minor surface irregularities and internal voids that may contribute to reduced mechanical performance. CUR release exhibited a sustained, concentration-dependent profile, with higher cumulative release from 10 mg CUR MNs and more stable release from 2 mg CUR MNs, indicating improved matrix retention at lower loading. Overall, moderate CUR loading (F2) combined with optimized geometric spacing produced MNs with balanced mechanical integrity, structural fidelity, and controlled release behavior suitable for microneedle-based delivery platforms.

## Figures and Tables

**Table 1:** CUR dosage per formulation and patch.

No.	Drug (mg)	Total Volume (mL)	Curcumin Dosage (µg/mL)	Patch Volume (mL)	Curcumin Dosage per Patch (µg)
F1	0	270	0	0.344	0
F2	2	270	7.41	0.344	2.55
F3	4	270	14.81	0.344	5.10
F4	6	270	22.22	0.344	7.64
F5	8	270	29.63	0.344	10.19
F6	10	270	37.04	0.344	12.74

**Table 2:** Tensile mechanical properties of PVA/PVP-CUR microneedle material.

F#	UTS (MPa)	Young’s Modulus (MPa)	Yield Strength (MPa)	Strain at Fmax (%)
F1	4.8133	2.8909	2.3526	166.5000
F2	4.0133	2.2067	2.2248	181.8667
F3	3.7367	1.9516	2.0226	191.4667
F4	3.4767	1.7407	1.8203	199.7267
F5	2.8267	1.2837	1.7671	220.2000
F6	2.4833	0.9931	1.5116	250.0667

**Table 3:** Results of FEA simulation on selected MN models (labeled A, B, C, and D).

Conical					
Array	Model	CUR (mg)	Total Deformation (mm)	Equivalent Stress (MPa)	Factor of Safety
11 × 11		0	0.2942	0.6244	3.768
	A	2	0.3854		3.563
	B	4	0.4358		3.239
	C	6	0.4885		2.915
13 × 13		0	0.3136	0.8124	2.896
	D	2	0.4109		2.739

**Table 4:** Penetration efficiency of different PVA/PVP-CUR microneedle arrays.

Model	Number of MNs on Array	Mean ± SD (Visible Blue-Stained Punctures)	Penetration Efficiency (%)
A	121	117 ± 2.52	96.69
B	121	112 ± 6.43	92.56
C	121	117 ± 2.31	96.69
D	169	160 ± 4.73	94.67

**Table 5:** Insertion efficiency of different PVA/PVP-CUR microneedle arrays.

Model	Initial Height (µm)	Mean Insertion Depth (µm)	Insertion Efficiency (%)
A	1500	1220	81.33
B	1500	1190	79.33
C	1500	1201	80.07
D	1500	960	80.83

## Data Availability

The original contributions presented in this study are included in the article/Supplementary Material. Further inquiries can be directed to the corresponding author(s).

## Supplementary Materials

The following supporting information can be downloaded at: https://www.mdpi.com/article/10.3390/polym18151912/s1, Table S1: PVA-PVP-CUR formulation; Figure S1: 3D models of microneedle designs showing top and side views of (a) conical, (b) pyramidal, and (c) cylindrical tip geometries; Figure S2: Microneedle features; Table S2. Microneedle geometrical parameters and array configurations; Table S3: Elimination criteria; Figure S3: Conical microneedle model in (a) array and (b) close-up view designed on Autodesk Fusion 360; Table S4: Conical MN dimensions; Figure S4: Pyramidal microneedle model in (a) array and (b) close-up view designed on Autodesk Fusion 360; Table S5: Pyramidal MN dimensions; Figure S5: Bulleted microneedle model in (a) array and (b) close-up view designed on Autodesk Fusion 360: Table S6: Bulleted MN dimensions; Figure S6: 3D view of microneedle array geometries (a) conical, (b) pyramidal, and (c) bulleted models on ANSYS software; Table S7: ANSYS results for conical MN models; Table S8: ANSYS results for pyramidal MN models; Table S9: ANSYS results for bulleted MN models; Figure S7: Total deformation and equivalent stress for 11 × 11 conical (0 mg CUR); Figure S8: Total deformation and equivalent stress for 11 × 11 conical (2 mg CUR); Figure S9: Total deformation and equivalent stress for 11 × 11 conical (4 mg CUR); Figure S10: Total deformation and equivalent stress for 11 × 11 conical (6 mg CUR); Figure S11: Total deformation and equivalent stress for 13 × 13 conical (0 mg CUR); Figure S12: Total deformation and equivalent stress for 13 × 13 conical (2 mg CUR); Figure S13: Pre-insertion images of models; Figure S14: Post insertion images of models; Figure S15: SEM (300× magnification) images of (a) drug-free and (b) drug-filled microneedles; Figure S16: FT-IR spectra of polymer components.
