# Supplementary material for: Fabrication, Simulation, and Mechanical Characterization of Curcumin-Loaded PVA/PVP Microneedle Arrays Using Custom 3D-Printed Molds
Source: Polymers (Basel). 2026 Aug 4;18(15):1912. doi: 10.3390/polym18151912 (PMC13468145; doi:10.3390/polym18151912)
Supplement: Supplementary file 1 [file polymers-18-01912-s001.zip › polymers-4038971-supplementary.pdf]

Supplementary Data

Table S1. PVA-PVP-CUR formulation.

No.	CUR (mg)	ETHA (ml)	PVA (g)	PVP (g)	Sorbitol 5 % w/w
**F1**	0	2	18	6	5
**F2**	2	2	18	6	5
**F3**	4	2	18	6	5
**F4**	6	2	18	6	5
**F5**	8	2	18	6	5
**F6**	10	2	18	6	5

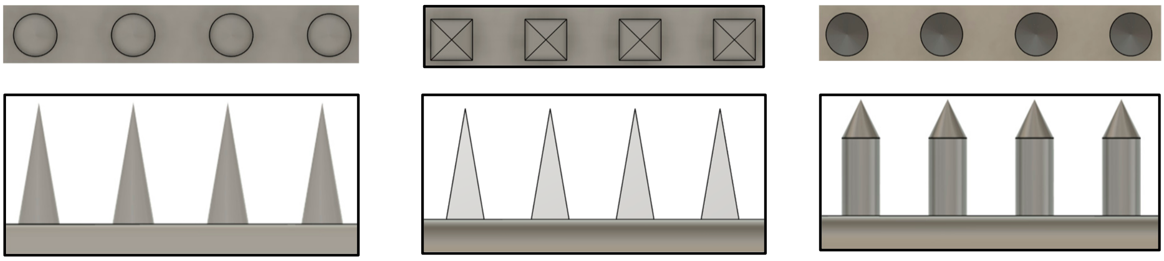

(a) (b) (c)

Figure S1. 3D models of microneedle designs showing top and side views of (a) conical, (b) pyramidal, and (c) cylindrical tip geometries.

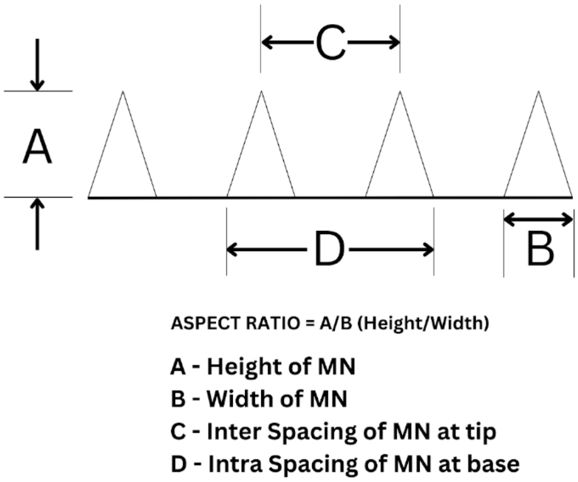

Figure S2. Microneedle features.

Table S2. Microneedle geometrical parameters and array configurations.

Height (µm)	Value	Inter-Spacing (µm)	Intra-Spacing (µm)	Array
1500	500	1125	1625	11 x 11
1200	400	900	1300	13 x 13
900	300	675	975	15 x 15

Table S3. Elimination criteria.

Criterion	Threshold	Reference
Total Deformation	< 75% of the needle height	[117]
Equivalent Stress	< 80% of yield strength ÷ 2	[118]
Factor of Safety	> 1.5	[120]

(A) 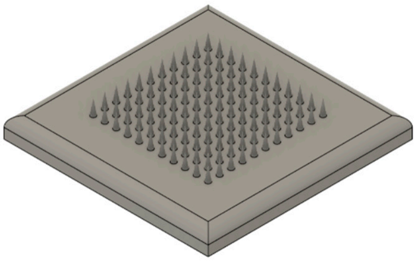

(B) 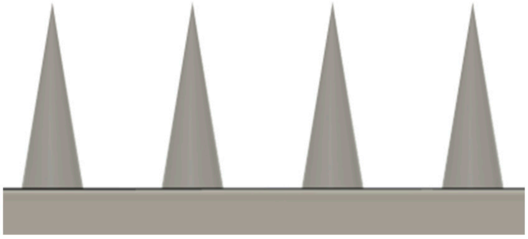

Figure S3. Conical microneedle model in (A) array and (B) close-up view designed on Autodesk Fusion 360.

Table S4. Conical MN dimensions.

Conical MN#	Height (µm)	Base Diameter (µm)	Inter-Spacing (µm)	Intra-Spacing (µm)	Array
1	1500	500	1125	1625	11x11
2	1200	400	900	1300	13x13
3	900	300	675	975	15x15

(A) **(B)**

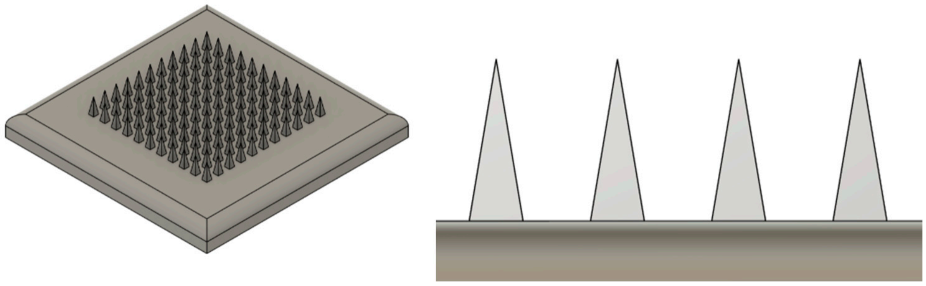

Figure S4. Pyramidal microneedle model in (A) array and (B) close-up view designed on Autodesk Fusion 360.

Table S5. Pyramidal MN dimensions.

Pyramidal MN #	Height (µm)	Base Diameter (µm)	Inter-Spacing (µm)	Intra-Spacing (µm)	Array
1	1500	500	1125	1625	11x11
2	1200	400	900	1300	13x13
3	900	300	675	975	15x15

(A) **(B)**

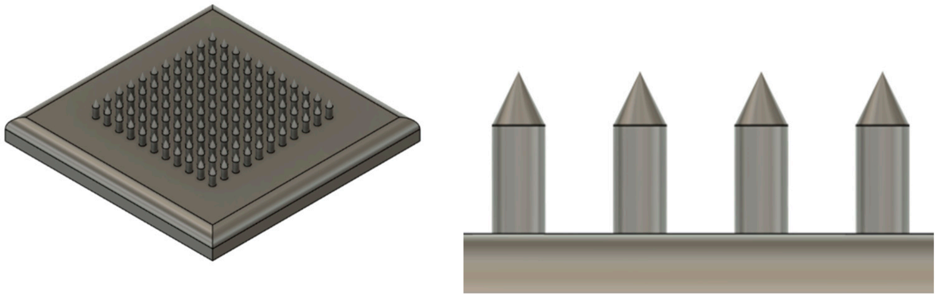

Figure S5. Bulleted microneedle model in (A) array and (B) close-up view designed on Autodesk Fusion 360.

Table S6. Bulleted MN dimensions.

Bulleted MN#	Height (µm)	Base Diameter (µm)	Inter-Spacing (µm)	Intra-Spacing (µm)	Array (µm)
1	1500	500	1125	1625	11x11
2	1200	400	900	1300	13x13
3	900	300	675	975	15x15

(A) **(B)** **(C)**

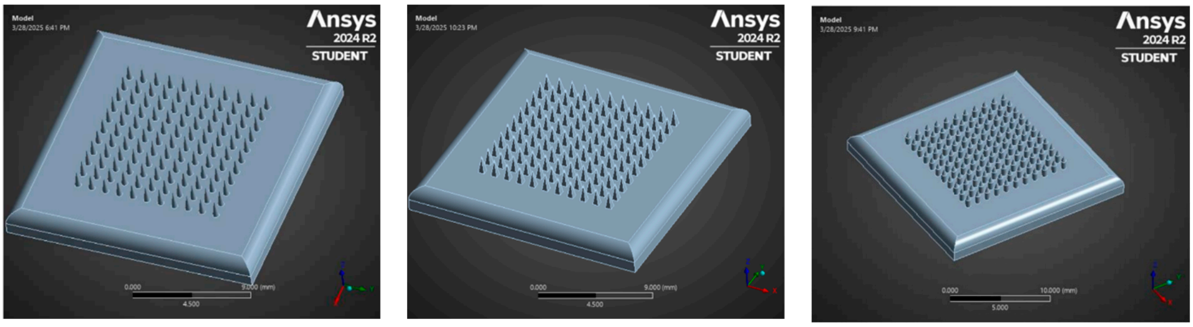

Figure S6. 3D view of microneedle array geometries: (A) conical, (B) pyramidal, and (C) bulleted models on ANSYS software.

Table S7. ANSYS results for conical MN models.

Microneedle Geometry	Array	CUR (mg)	Total Deformation (mm)	Equivalent Stress (MPa)	Factor of Safety	Elimination Criteria Met?
	11x11	0	0.2942	0.6244	3.768	Yes
		2	0.3854		3.563	Yes
		4	0.4358		3.239	Yes
		6	0.4885		2.915	Yes
		8	0.6625		2.387	No
		10	0.8563		2.370	No

**Conical**	13x13	0	0.3136	0.8124	2.896	Yes
		2	0.4109		2.739	Yes
		4	0.4646		2.490	No
		6	0.5209		2.241	No
		8	0.7063		1.834	No
		10	0.9130		1.821	No
	15x15	0	0.2856	0.9819	2.396	No
		2	0.3741		2.266	No
		4	0.4230		2.060	No
		6	0.4743		1.854	No
		8	0.6431		1.518	No
		10	0.8313		1.507	No

Table S8. ANSYS results for pyramidal MN models.

Microneedle Geometry	Array	CUR (mg)	Total Deformation (mm)	Equivalent Stress (MPa)	Factor of Safety	Elimination Criteria Met?
	11x11	0	2.9014	21.7840	0.108	No
		2	3.8010		0.102	No
		4	4.2979		0.093	No
		6	4.8186		0.084	No
		8	6.5341		0.068	No
		10	8.4461		0.068	No
	13x13	0	1.2573	8.2818	0.379	No
		2	1.6472		0.358	No
		4	1.8625		0.326	No
		6	2.0881		0.293	No

Pyramidal		8	2.8315		0.240	No
		10	3.6600		0.238	No
	15x15	0	1.2704	13.3250	0.177	No
		2	1.6643		0.167	No
		4	1.8818		0.152	No
		6	2.1098		0.137	No
		8	2.8609		0.112	No
		10	3.6981		0.111	No

Table S9. ANSYS results for bulleted MN models.

Microneedle Geometry	Array	CUR (mg)	Total Deformation (mm)	Equivalent Stress (MPa)	Factor of Safety	Elimination Criteria Met?
Bulleted	11x11	0	1.4437	13.3250	0.177	No
		2	1.8913		0.167	No
		4	2.1386		0.152	No
		6	2.3977		0.137	No
		8	3.2513		0.112	No
		10	4.2026		0.111	No
	13x13	0	0.7944	8.1485	0.289	No
		2	1.0407		0.273	No
		4	1.1767		0.248	No
		6	1.3193		0.223	No
		8	1.7889		0.183	No
		10	2.3124		0.182	No
		0	0.8180		0.217	No
		2	1.0716		0.205	No

	15x15	4	1.2117	10.8660	0.186	No
		6	1.3585		0.168	No
		8	1.8421		0.137	No
		10	2.3811		0.136	No

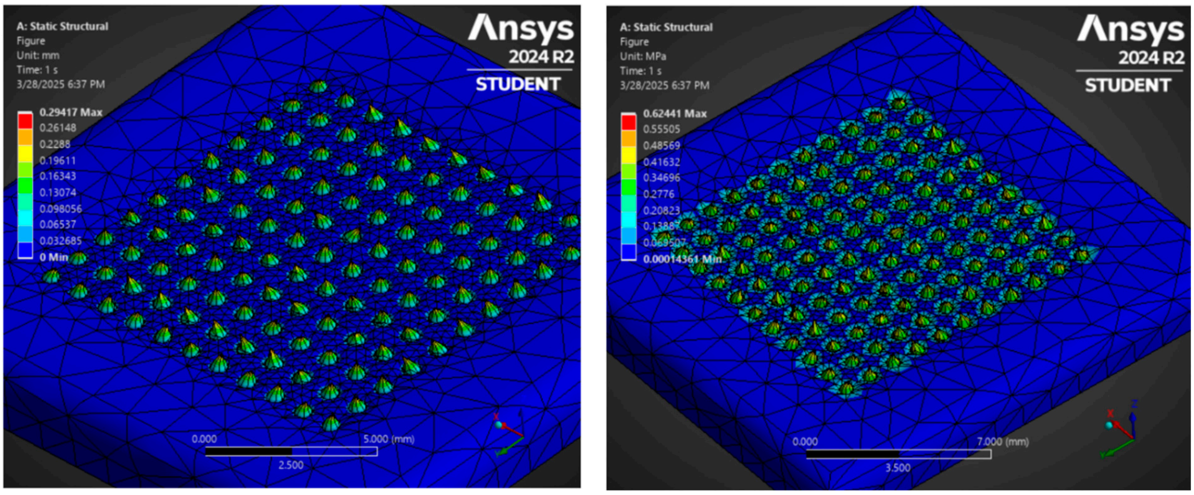

Figure. S7. Total deformation and equivalent stress for 11 x 11 conical (0 mg CUR).

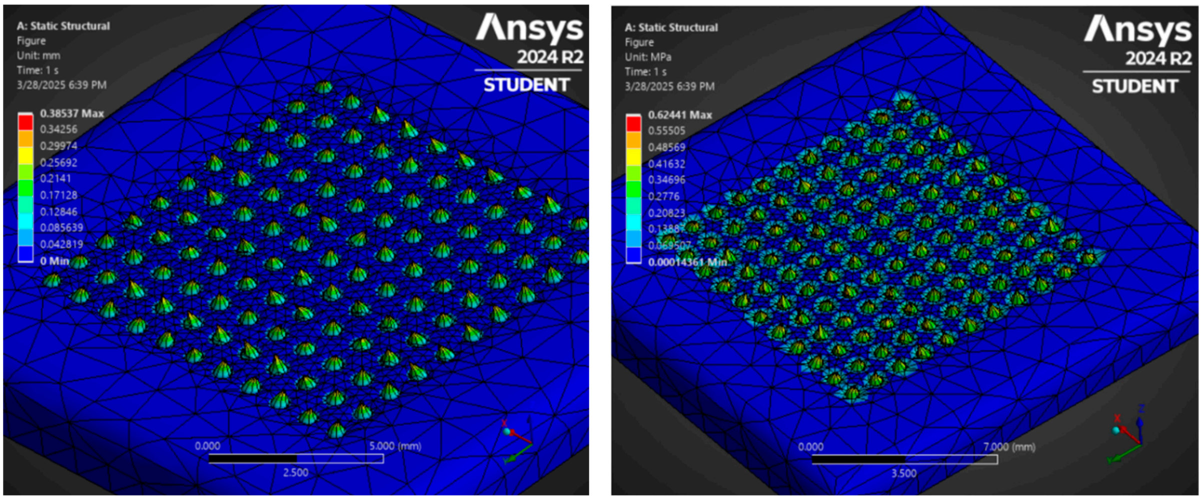

Figure S8. Total deformation and equivalent stress for 11 x 11 conical (2 mg CUR).

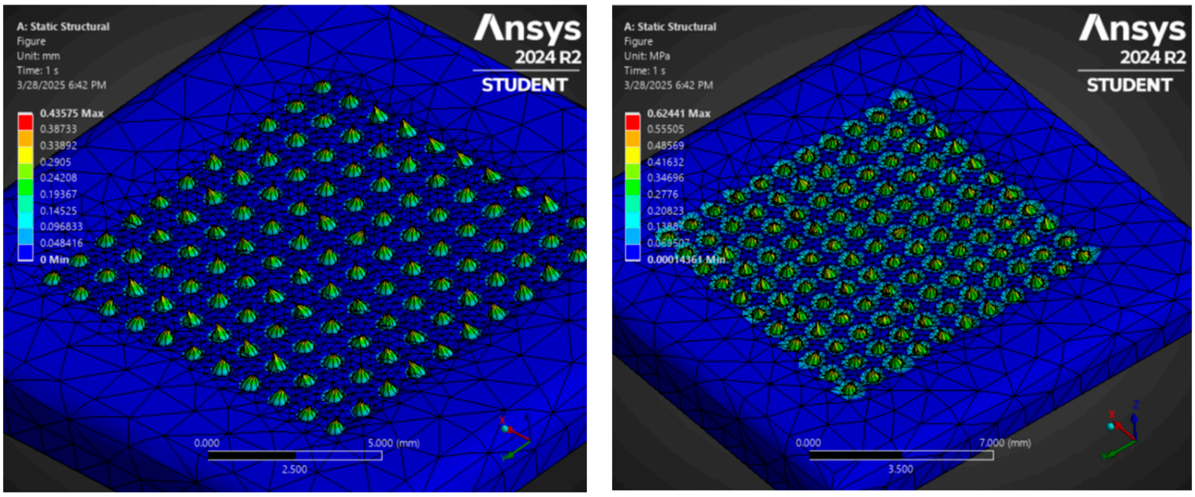

Figure S9. Total deformation and equivalent stress for 11 x 11 conical (4 mg CUR).

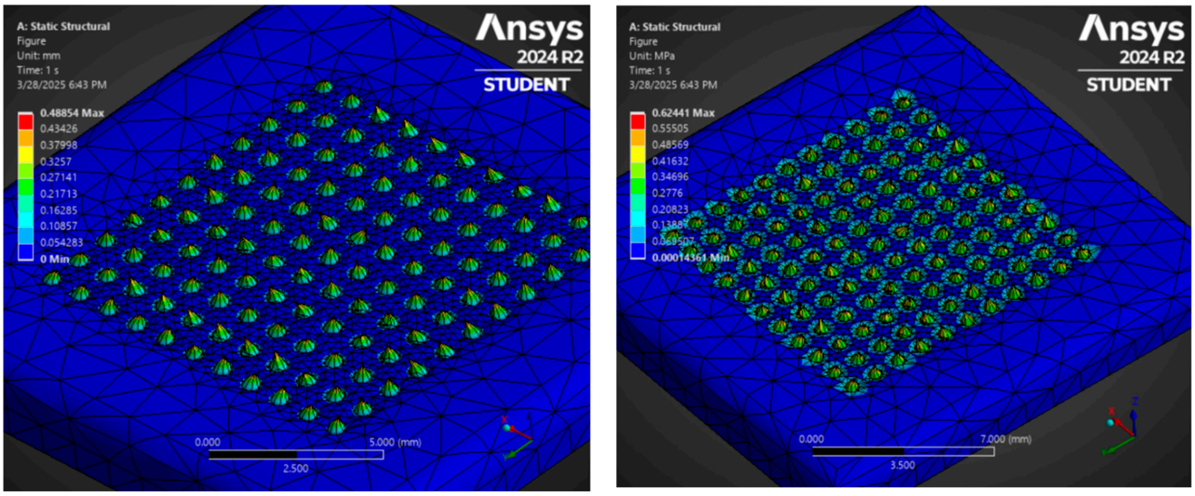

Figure S10. Total deformation and equivalent stress for 11 x 11 conical (6 mg CUR).

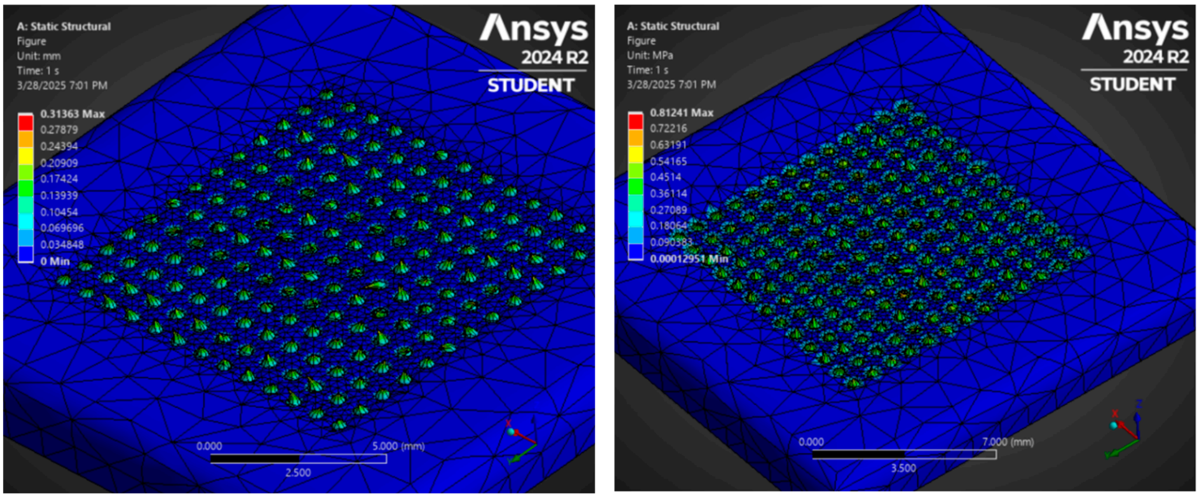

Figure S11. Total deformation and equivalent stress for 13 x 13 conical (0 mg CUR).

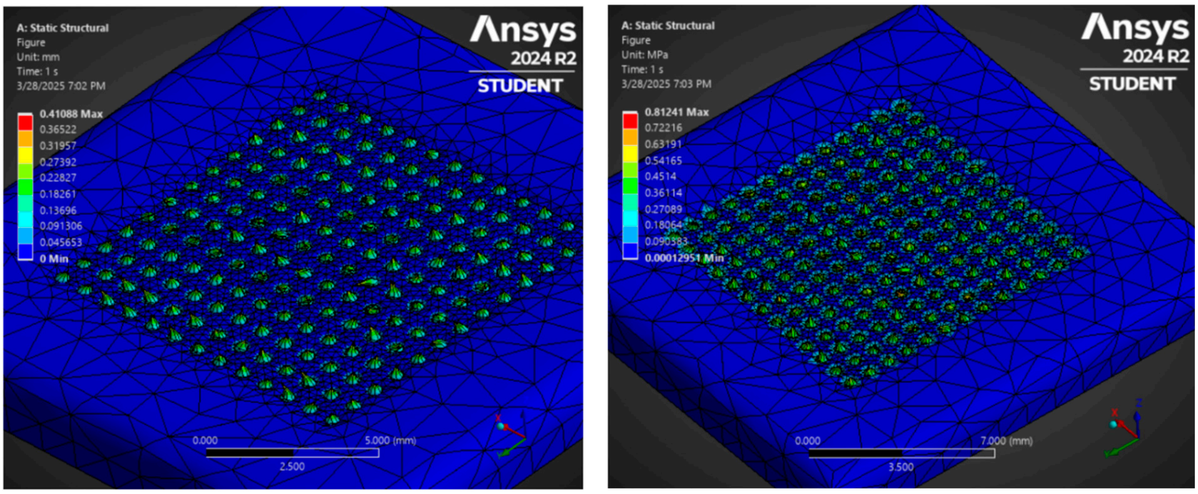

Figure S12. Total deformation and equivalent stress for 13 x 13 conical (2 mg CUR).

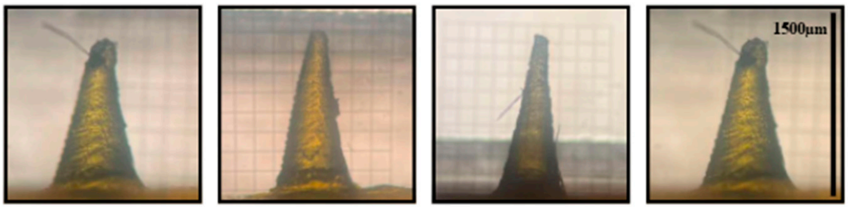

(A) (B) (C) (D)

Figure S13. Pre-insertion images of models.

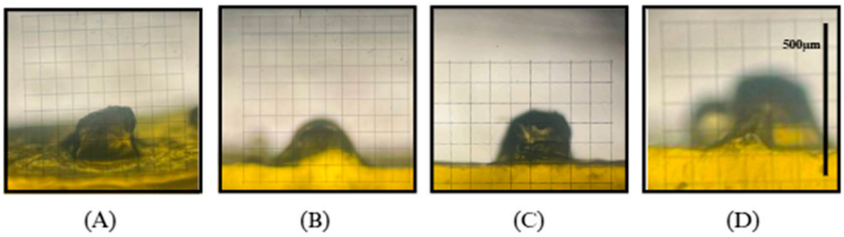

Figure S14. Post-insertion images of models.

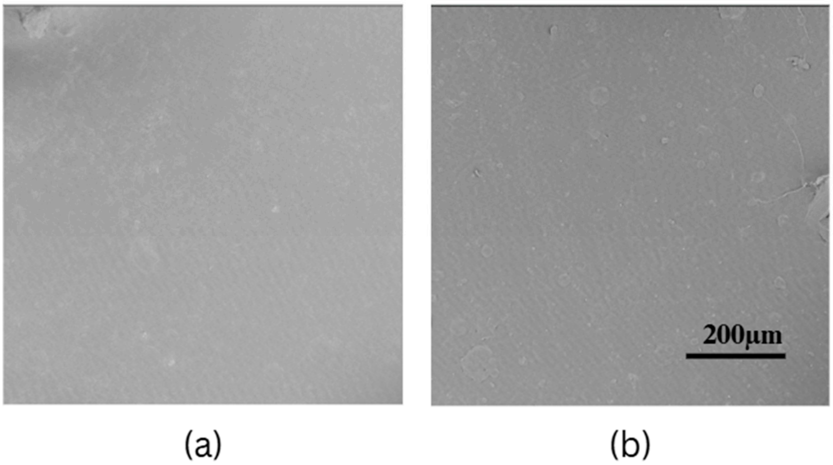

Figure S15. SEM (300x magnification) images of (a) drug-free and (b) drug-filled microneedles.

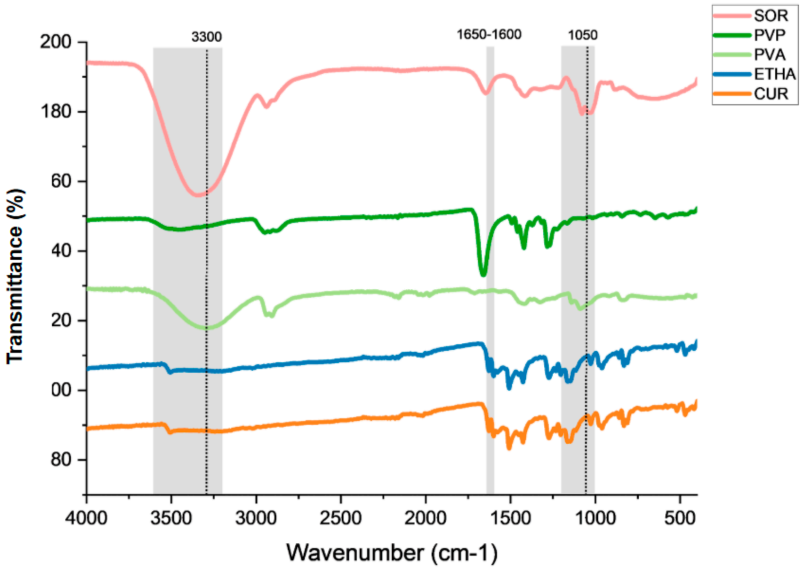

Figure S16. FT-IR spectra overlay of polymer components.
